# Effect of Telehealth Services on Mitral and Tricuspid Regurgitation Progression: Retrospective Study

**DOI:** 10.2196/47947

**Published:** 2023-09-26

**Authors:** Li-Tan Yang, Jen-Kuang Lee, Chieh-Mei Tsai, Ying-Hsien Chen, Ching-Chang Huang, Hui-Wen Wu, Chin-Hua Su, Chien-Chang Lee, Chi-Sheng Hung, Yi-Lwun Ho

**Affiliations:** 1 Division of Cardiology Department of Internal Medicine National Taiwan University Hospital Taipei Taiwan; 2 Department of Internal Medicine College of Medicine National Taiwan University Taipei Taiwan; 3 Telehealth Center National Taiwan University Hospital Taipei Taiwan; 4 Department of Emergency Medicine National Taiwan University Hospital Taipei Taiwan; 5 Division of Cardiology, Department of Internal Medicine National Taiwan University Hospital Taipei Taiwan

**Keywords:** mitral regurgitation, tricuspid regurgitation, telehealth, telemedicine, point-of-care ultrasound, cardiovascular health, heart disease, cardiac, cardiology, patience care, patient-reported outcome, remote monitoring, health management

## Abstract

**Background:**

Mitral regurgitation (MR) and tricuspid regurgitation (TR) are common cardiac conditions with high mortality risks, which can be improved through early intervention. Telehealth services, which allow for remote monitoring of patient conditions, have been proven to improve the health management of chronic diseases, but the effects on MR and TR progression are unknown.

**Objective:**

This study aimed to explore whether patients receiving telehealth services have less MR and TR progression compared with a control group. We also aimed to identify the determinants of MR and TR progression.

**Methods:**

This single-center retrospective study conducted at the National Taiwan University Hospital compared MR and TR progression (defined as either progression to moderate or greater MR and TR or MR and TR progression by ≥2 grades during the study period) between the telehealth and control groups. Patients had a minimum of 2 transthoracic echocardiograms at least 6 months apart; baseline mild-moderate MR and TR or lower; and no prior surgeries on the mitral or tricuspid valve. Telehealth patients were defined as those who received telehealth services for at least 28 days within 3 months of baseline. Basic demographics, baseline blood pressure measurements, prescribed medication, and Charlson Comorbidity Index components were obtained for all patients.

**Results:**

A total of 1081 patients (n=226 in the telehealth group and n=855 in the control group) were included in the study analyses. The telehealth group showed significantly lower baseline systolic blood pressure (*P*<.001), higher Charlson Comorbidity Index (*P*=.02), higher prevalence of prior myocardial infarction (*P*=.01) and heart failure (*P*<.001), higher beta-blocker (*P*=.03) and diuretic (*P*=.04) use, and lower nitrate use (*P*=.04). Both groups showed similar cardiac remodeling conditions at baseline. Telehealth was found to be neutral for both MR (hazard ratio 1.10, 95% CI 0.80-1.52; *P*=.52) and TR (hazard ratio 1.27, 95% CI 0.92-1.74; *P*=.14) progression. Determinants for moderate or greater MR progression included older age, female sex, diuretic use, larger left atrial dimension, left ventricular end-diastolic dimension, left ventricular end-systolic dimension, and lower left ventricular ejection fraction. Determinants of moderate or greater TR progression included older age, female sex, diuretic use, presence of atrial fibrillation, LA dimension, left ventricular end-systolic dimension, and lower left ventricular ejection fraction; statin use was found to be protective.

**Conclusions:**

This is the first study to assess the association between telehealth services and the progression of MR and TR. Telehealth patients, who had more comorbidities, displayed similar MR and TR progression versus control patients, indicating that telehealth may slow MR and TR progression. Determinants of MR and TR progression included easy-to-measure traditional echo parameters of cardiac function, older age, female sex, and atrial fibrillation, which can be incorporated into a telehealth platform and advanced alert system, improving patient outcomes through personalized care.

## Introduction

Mitral regurgitation (MR) has been reported to be the most common type of valvular heart disease, and severe conditions are associated with poor cardiac outcomes, hospitalization, and mortality risks [1-3]. MR prevalence has increased in recent years, and early intervention or surgery often results in good long-term outcomes and prolonged survival [1,3,4], necessitating early detection and monitoring of this condition. Tricuspid regurgitation (TR) is also a common echocardiographic finding; it has been reported that up to 15% of patients referred for echocardiography have been found to have moderate or severe TR [5,6]. Similarly, TR is associated with poor cardiac outcomes and heightened mortality risks [6,7].

Due to their association with poor prognoses, both MR and TR have received increasing attention in recent years. Given the increasing burdens of these conditions and the benefits of early intervention, a clear understanding of clinical factors that accelerate MR and TR progression can help to identify patient groups that require close monitoring.

In the past, telehealth services for self-management of chronic conditions were largely concentrated on patient education, remote consultations, and lifestyle interventions [8], but the heightened demand for telehealth triggered by the COVID-19 pandemic and lockdown policies has triggered great advances in remote patient monitoring technologies [9]. Previous studies have shown that telemedicine can effectively reduce rehospitalization and mortality of patients with heart failure, possibly due to improved drug compliance, better control of cardiovascular factors, and early detection of adverse conditions [10]. However, the association between telemedicine and MR or TR progression remains unclear. We hypothesized that participation in telehealth services may slow MR and TR progression. In this study, we aimed to explore (1) whether telemedicine was linked to slower MR and TR progression compared with the control group and (2) the determinants of MR and TR progression.

## Methods

### Ethics Approval

This single-center retrospective study obtained approval from the Institutional Review Board of National Taiwan University Hospital (NTUH) in Taipei, Taiwan (201804072RINA) and was conducted by the Taiwan ELEctroHEALTH study group (TELEHEALTH study group). Written consent was obtained from all participants prior to the study.

### Telehealth Services

The Telehealth Center of NTUH has provided telehealth services targeted to patients with cardiovascular disease since 2009. Patients diagnosed by and admitted to the cardiovascular ward at NTUH, who had multiple cardiovascular disease risk factors or established conditions such as arrhythmias, myocardial infarction (MI), coronary artery disease, congestive heart failure, or other surgical or congenital heart conditions were invited to enroll in our telehealth program. Our control group included subjects who were admitted to our cardiovascular center during the same period but declined to participate in the telehealth care program and received usual care only.

Participating patients were first evaluated for telehealth eligibility before they and their main caregivers received a face-to-face tutorial where they were instructed on how to operate all telehealth devices (manometer, oximeter, glucometer, and electrocardiography devices). In particular, patients were taught to measure blood pressure (BP) at home based on guideline suggestions [11,12] using commercially available BP machines. Patients’ internet access and biometrics transmission ability were confirmed via a home visit before home telehealth services were launched.

Patients’ biometric data, including BP, pulse rate, finger-stick glucose, single-lead electrocardiography, and oxygen saturation were measured daily or on demand and transmitted to a cloud database developed by the Graduate Institute of Biomedical Electronics and Bioinformatics, National Taiwan University, Taiwan [13] to be monitored and reviewed by case managers or physicians, who discussed patient conditions and provide clinical suggestions as necessary. Our case managers processed all patient data immediately upon receipt. If measurement values were too low, too high, or had any other issues, the case manager would immediately call the patient to confirm their condition. For example, if high BP was detected, the case manager would check if the patient took medication or if there were other reasons which led to elevated BP.

The data transmission process and subsequent handling by our Telehealth Center are shown in Figure S1 in Multimedia Appendix 1. Previous studies have detailed enrollment criteria and scope of services for our telehealth services [14-17], which we will not reiterate here.

### Study Population

**Figure 1:**
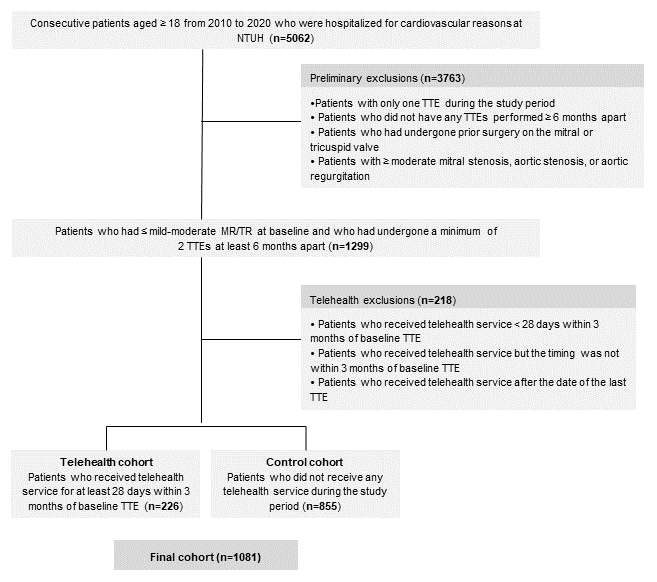
Study flowchart.

Our study flowchart is shown in Figure 1. This study included patients who were hospitalized at NTUH for cardiovascular reasons between 2010 and 2020. We obtained data for a total of 5062 patients; of these, 2537 were enrolled in our telehealth services and 2525 belonged to our control group.

Study inclusion criteria were (1) patients who had undergone at least 2 transthoracic echocardiograms (TTEs) that were at least 6 months apart; (2) no presence of greater than or equal to moderate MR or TR at baseline TTE; (3) no prior surgery on the mitral or tricuspid valve; (4) and no greater than or equal to moderate mitral stenosis, aortic stenosis, or aortic regurgitation. After exclusion, we classified the remaining patients into the telehealth group and the control group. The telehealth group was defined as those who received telehealth services for at least 28 days within 3 months of baseline TTE. The control group was defined as those who never had received telehealth services. To avoid confounding, we also excluded patients who were enrolled in telehealth services for less than 28 days within 3 months of baseline TTE or who received telehealth services after the last available TTE.

### Clinical Data

We obtained basic demographics, baseline BP measurements, prescribed medication, and comorbid conditions for all patients; the Charlson Comorbidity Index (CCI) was derived without information on acquired immunodeficiency syndrome status, which was kept confidential in accordance with the HIV Infection Control and Patient Rights Protection Act.

### Primary End Point

The primary end point of this study was MR or TR progression, defined as (1) progression from baseline less than moderate MR or TR to greater than or equal to moderate MR or TR and above; or (2) MR or TR progression by greater than or equal to 2 grades as compared with baseline TTE during the study period. MR and TR were graded from 0 to 6 as no, trivial, mild, mild-moderate, moderate, moderate-severe, and severe.

### Echocardiography

The first eligible TTE was used as the baseline for analysis in patients with multiple studies. TTE was performed by trained sonographers using commercially available echo systems. For the primary end point of progression from baseline less than moderate MR or TR to greater than or equal to moderate MR or TR and above (progressors), the first TTE showing greater than or equal to moderate MR or TR (index TTE) was used, and the follow-up time was set as the period between baseline and index TTEs. For the end point of MR or TR progression by 2 grades or more (progressors), the first TTE showing MR or TR progression to greater than or equal to 2 grades (index TTE) was used, and the follow-up time was set as the period between baseline and index TTEs. For nonprogressors, the follow-up time was between the baseline and the last eligible TTE. For patients who underwent cardiac surgeries, only TTEs undertaken before the first surgery were included in our analyses. Chamber quantification was performed based on guideline suggestions [18]. The severity of MR and TR was graded via eyeball assessment and semiquantitative measurements by cardiologists in NTUH.

### Statistical Analysis

Continuous variables, expressed as mean (SD) or median (IQR) according to data distribution, were compared using *t* tests. Categorical data, presented as counts and percentages, were compared using chi-squared tests and Fisher exact test. The primary end point of MR or TR progression was analyzed using the Cox-proportional hazard model, where variables with clinical relevance plus univariate *P*<.05 were chosen for multivariable analyses. Left ventricular ejection fraction (LVEF), left ventricular end-diastolic dimension (LVEDD), and left ventricular end-systolic dimension (LVESD) were placed in different models due to colinearity (Pearson *r* of LVEF with LVEDD and LVESD were −0.51 and −0.86, respectively). Statistical analyses were performed using a combination of commercially available software (JMP 16 and SAS 9.4; SAS Institute Inc) and the R software package (R Foundation for Statistical Computing). A 2-sided *P*<.05 was considered statistically significant.

## Results

### Baseline Characteristics

A total of 1081 patients (n=226 in the telehealth group and n=855 in the control group) were included in the study analyses. Patient baseline characteristics are shown in Table 1. Compared to the telehealth group, the control group had higher baseline systolic BP (mean 123, SD 16 vs mean 133, SD 21 mm Hg; *P*<.001), lower CCI scores (mean 1.41, SD 1.7 vs mean 1.10, SD 1.40; *P=*.02), lower prevalence of prior MI (37/226, 16% vs 86/855, 10%; *P=*.01) and heart failure history (51/226, 23% vs 100/855, 12%; *P<*.001), lower prevalence of beta-blocker (165/226, 73% vs 559/855, 65%; *P=*.02) and diuretic (99/226, 44% vs 311/855, 36%; *P=*.04) use, and higher prevalence of nitrate use (91/226, 40% vs 408/855, 48%; *P=*.04). The telehealth group had similar cardiac remodeling conditions as compared with the control group signified by left atrial (LA) dimension, LVEDD, LVESD, and LVEF, as well as similar baseline MR and TR severity.

**Table 1:** Baseline characteristics between telehealth, partial telehealth group, and control group (n=1273).

			Telehealth (n=226)		Control (n=855)		*P* value	
**Demographics**								
	Age (years), mean (SD)	63 (15)		64 (14)		.15		
	Male, n (%)	173 (77)		609 (71)		.10		
	SBP^a^ (mm Hg), mean (SD)	123 (16)		133 (21)		*<.001* ^b^		
	DBP^c^ (mm Hg), mean (SD)	74 (12)		74 (14)		.31		
	Atrial fibrillation, n (%)	6 (3)		27 (3)		.69		
	Charlson Comorbidity Index, mean (SD)	1.41 (1.7)		1.10 (1.40)		*.02*		
	Hypertension, n (%)	102 (45)		443 (52)		.07		
	Diabetes mellitus, n (%)	55 (24)		253 (30)		.11		
	Myocardial infarction, n (%)	37 (16)		86 (10)		*.01*		
	Heart failure history, n (%)	51 (23)		100 (12)		*<.001*		
	Malignancy, n (%)	18 (8)		60 (7)		.62		
**Medications, n (%)**								
	Statin	113 (50)		429 (50)		.96		
	Antiplatelet	155 (69)		640 (75)		.06		
	Alpha blocker	36 (16)		106 (12)		.17		
	ACEI^d^ and ARB^e^	137 (61)		478 (56)		.20		
	Beta-blocker	165 (73)		559 (65)		*.02*		
	Calcium channel blocker	94 (42)		336 (39)		.53		
	Diuretics	99 (44)		311 (36)		*.04*		
	Nitrate	91 (40)		408 (48)		*.04*		
**Baseline cardiac chamber size, mean (SD)**								
	Left ventricular ejection fraction (%)	63 (13)		63 (13)		.86		
	LA^f^ dimension (cm)	3.7 (0.6)		3.7 (0.6)		.79		
	LVEDD^g^ (mm)	4.8 (0.6)		4.8 (0.6)		.23		
	LVESD^h^ (mm)	3.2 (0.8)		3.2 (0.8)		.55		
**Baseline MR^i^, n (%)**								.08
	None	17 (8)		78 (9)				
	Trivial	17 (8)		66 (8)				
	Mild	142 (63)		584 (68)				
	Mild-moderate	50 (22)		127 (15)				
**Baseline TR^j^, n (%)**								.30
	None	18 (8)		83 (10)				
	Trivial	28 (12)		91 (11)				
	Mild	142 (63)		572 (67)				
	Mild-moderate	38 (17)		109 (13)				
**Maximal MR during follow-up, n (%)**								.63
	None	5 (2)		9 (1)				
	Trivial	5 (2)		19 (2)				
	Mild	124 (55)		461 (54)				
	Mild-moderate	43 (19)		178 (21)				
	Moderate	46 (20)		167 (20)				
	Moderate-severe	3 (1)		17 (2)				
	Severe	0 (0)		4 (<1)				
	Maximal MR greater than or equal to moderate	49 (22)		188 (22)		.92		
	Maximal MR greater than or equal to 2 grades compared to baseline	49 (22)		204 (24)		.48		
**Maximal TR during follow-up, n (%)**								.47
	None	1 (<1)		4 (<1)				
	Trivial	1 (<1)		14 (2)				
	Mild	123 (54)		456 (53)				
	Mild-moderate	50 (22)		173 (20)				
	Moderate	47 (21)		179 (21)				
	Moderate-severe	4 (2)		25(3)				
	Severe	0 (0)		4 (<1)				
	Maximal TR greater than or equal to moderate	49 (22)		188 (22)		.92		
	Maximal TR greater than or equal to 2 grades compared to baseline	47 (21)		235 (27)		*.03*		

^a^SBP: systolic blood pressure.

^b^Italic formatting represents *P* value<.05.

^c^DBP: diastolic blood pressure.

^d^ACEI: angiotensin-converting enzyme inhibitor.

^e^ARB: angiotensin receptor blocker.

^f^LA: left atrium.

^g^LVEDD: left ventricular end-diastolic dimension.

^h^LVESD: left ventricular end-systolic dimension.

^i^MR: mitral regurgitation.

^j^TR: tricuspid regurgitation.

### Determinants of MR Progression to Greater Than or Equal to Moderate Degree

At a median follow-up of 4.6 (IQR 2.2-7) years, 237 out of 1081 patients had MR progression to a greater than or equal to moderate degree. Univariate determinants of progression to moderate MR and above included: age, sex, CCI, presence of atrial fibrillation (AF), LVEF, LVEDD, LVESD, LA size, statin use, diuretics, and angiotensin-converting enzyme inhibitors and angiotensin receptor blockers (ACEI and ARB; all *P*≤.03); the telehealth group was not associated with MR progression (hazard ratio [HR] 1.10, 95% CI 0.80-1.52; *P*=.52). Table 2 shows the final multivariate models for determinants of progression to moderate MR and above. Older age, female sex, diuretics use, larger LA, lower LVEF, and larger LVEDD and LVESD were associated with progression to moderate MR and above (all *P*≤.02). Interestingly, statin use appeared to be protective against MR progression.

**Table 2:** Multivariate determinants of mitral regurgitation progression in patients with less than or equal to mild-moderate mitral regurgitation at baseline: end point—mitral regurgitation progression to greater than or equal to moderate degree (n=237).

	LVEF^a^ model		LVEDD^b^ model			LVESD^c^ model	
	HR^d^ (95% CI)	*P* value	HR (95% CI)	*P* value	HR (95% CI)		*P* value
Age	1.02 (1.01-1.03)	*<.001* ^e^	1.02 (1.01-1.03)	*<.001*	1.02 (1.01-1.03)		*<.001*
Female	1.73 (1.29-2.32)	*<.001*	1.74 (1.29-2.33)	*<.001*	1.77 (1.31-2.37)		*<.001*
SBP^f^ (mm Hg)	1.00 (0.99-1.00)	.76	0.99 (0.99-1.00)	.96	1.00 (0.99-1.00)		.92
CCI^g^	1.04 (0.95-1.14)	.37	1.03 (0.94-1.12)	.47	1.03 (0.94-1.13)		.41
AF^h^	1.60 (0.85-3.03)	.16	1.91 (1.02-3.59)	.06	1.74 (0.92-3.29)		.10
ACEI^i^ and ARB^j^	1.13 (0.85-1.51)	.38	1.12 (0.84-1.49)	.43	1.13 (0.84-1.51)		.39
Diuretics	1.61 (1.19-2.16)	*.001*	1.80 (1.35-2.40)	*<.001*	1.65 (1.23-2.21)		*<.001*
Statin	0.66 (0.50-0.88)	*.003*	0.67 (0.51-0.89)	*.004*	0.67 (0.51-0.88)		*.004*
LAD^k^ (cm)	1.42 (1.13-1.78)	*.002*	1.36 (1.06-1.73)	*.01*	1.33 (1.05-1.68)		*.02*
LVEF (%)	0.97 (0.96-0.98)	*<.001*	N/A^l^	N/A	N/A		N/A
LVEDD (mm)	N/A	N/A	1.32 (1.07-1.61)	*.008*	N/A		N/A
LVESD (mm)	N/A	N/A	N/A	N/A	1.42 (1.22-1.63)		*<.001*

^a^LVEF: left ventricular ejection fraction.

^b^LVEDD: left ventricular end-diastolic dimension.

^c^LVESD: left ventricular end-systolic dimension.

^d^HR: hazard ratio.

^e^Italic formatting represents *P* value<.05.

^f^SBP: systolic blood pressure.

^g^CCI: Charlson Comorbidity Index.

^h^AF: atrial fibrillation.

^i^ACEI: angiotensin-converting enzyme inhibitors.

^j^ARB: angiotensin receptor blockers

^k^LAD: left atrium dimension.

^l^N/A: not available.

### Determinants of MR Progression to 2 Grades or More

At a median follow-up of 4.5 (IQR 2.1-7.0) years, 253 out of 1081 patients had MR progression to greater than or equal to 2 grades or more. Univariate determinants of MR progression by more than 2 grades included: age, sex, the presence of AF, LVEF, statin use, diuretics use, beta-blocker use, and antiplatelet use (all *P*≤.04). Again, the telehealth group was not associated with MR progression to greater than or equal to 2 grades (HR 1.00, 95% CI 0.73-1.38; *P*=.95). Table 3 shows the final multivariate models for determinants of MR progression to more than 2 grades, which included older age, female sex, and lower LVEF; the presence of AF showed a trend toward MR progression. Again, the use of diuretics appeared to be associated with MR progression.

**Table 3:** Multivariate determinants of mitral regurgitation progression in patients with less than or equal to mild-moderate mitral regurgitation at baseline: end point—increase of mitral regurgitation more than 2 grades (n=253).

	LVEF^a^ model			LVEDD^b^ model			LVESD^c^ model	
	HR^d^ (95% CI)	*P* value	HR (95% CI)		*P* value	HR (95% CI)		*P* value
Age	1.01 (1.00-1.02)	*<.001* ^e^	1.01 (1.00-1.02)		*.006*	1.01 (1.00-1.03)		*.001*
Female	1.66 (1.24-2.23)	*<.001*	1.55 (1.15-2.09)		*.003*	1.65 (1.23-2.21)		*.001*
SBP^f^ (mm Hg)	0.99 (0.99-1.00)	.48	0.99 (0.99-1.00)		.40	0.99 (0.99-1.00)		.43
CCI^g^	0.97 (0.87-1.07)	.60	0.96 (0.87-1.06)		.51	0.97 (0.87-1.06)		.56
AF^h^	1.82 (0.97-3.41)	.08	1.93 (1.03-3.62)		.05	1.92 (1.03-3.60)		.05
Antiplatelet	0.91 (0.65-1.27)	.59	0.88 (0.62-1.23)		.46	0.91 (0.65-1.28)		.59
Beta-blocker	0.92 (0.68-1.23)	.58	0.90 (0.67-1.22)		.52	0.91 (0.68-1.23)		.55
Diuretics	1.27 (0.95-1.70)	.09	1.41 (1.06-1.87)		*.02*	1.32 (0.99-1.76)		.05
Statin	0.80 (0.59-1.07)	.13	0.81 (0.60-1.08)		.16	0.81 (0.60-1.08)		.15
LAD^i^ (cm)	0.89 (0.70-1.12)	.35	0.93 (0.72-1.19)		.59	0.87 (0.68-1.11)		.28
LVEF (%)	0.98 (0.97-0.99)	*.005*	N/A^j^		N/A	N/A		N/A
LVEDD (mm)	N/A	N/A	0.95 (0.76-1.19)		.69	N/A		N/A
LVESD (mm)	N/A	N/A	N/A		N/A	1.16 (0.98-1.37)		.08

^a^LVEF: left ventricular ejection fraction.

^b^LVEDD: left ventricular end-diastolic dimension.

^c^LVESD: left ventricular end-systolic dimension.

^d^HR: hazard ratio.

^e^Italic formatting represents *P* value<.05.

^f^SBP: systolic blood pressure.

^g^CCI: Charlson Comorbidity Index.

^h^AF: atrial fibrillation.

^i^LAD: left atrium dimension.

^j^N/A: not available.

### Determinants of TR Progression to Greater Than or Equal to Moderate Degree

At a median follow-up of 4.7 (IQR 2.2-7.0) years, 237 out of 1081 patients had TR progression greater than or equal to moderate. Univariate determinants of progression to moderate TR and above included: age, female sex, CCI, the presence of AF, LVEF, LVEDD, LVESD, LA size, statin use, diuretics use, and antiplatelet use (all *P*≤.05). The telehealth group was not associated with TR progression (HR 1.27, 95% CI 0.92-1.74; *P*=.14). Table 4 shows the final multivariate models for determinants of progression to moderate TR and above. Older age, female sex, presence of AF, larger LA, lower LVEF, and larger LVESD were associated with progression to moderate TR and above (all *P*≤.04); larger LVEDD showed a trend toward TR progression. Statin use appeared to be protective, while the use of diuretics seemed to be associated with TR progression.

**Table 4:** Multivariate determinants of tricuspid regurgitation progression in patients with less than or equal to mild-moderate tricuspid regurgitation at baseline: end point—tricuspid regurgitation progression to greater than or equal to moderate degree (n=237).

	LVEF^a^ model		LVEDD^b^ model		LVESD^c^ model	
	HR^d^ (95% CI)	*P* value	HR (95% CI)	*P* value	HR (95% CI)	*P* value
Age	1.02 (1.01-1.03)	*<.001* ^e^	1.02 (1.01-1.03)	*<.001*	1.02 (1.01-1.03)	*<.001*
Female	2.04 (1.50-2.76)	*<.001*	1.99 (1.46-2.71)	*<.001*	2.07 (1.53-2.82)	*<.001*
SBP^f^	1.00 (0.99-1.00)	.58	1.00 (0.99-1.00)	.82	1.00 (0.99-1.00)	.73
CCI^g^	1.09 (0.99-1.20)	.06	1.08 (0.98-1.19)	.09	1.08 (0.99-1.19)	.08
AF^h^	1.91 (1.02-3.60)	.06	2.22 (1.18-4.15)	*.02*	2.05 (1.09-3.85)	*.04*
Antiplatelet	0.89 (0.63-1.24)	.50	0.86 (0.61-1.21)	.40	0.92 (0.66-1.30)	.66
Diuretics	1.56 (1.16-2.10)	*.002*	1.77 (1.32-2.36)	*<.001*	1.62 (1.21-2.17)	*.001*
Statin	0.64 (0.47-0.86)	*.003*	0.67 (0.49-0.90)	*.008*	0.65 (0.48-0.88)	*.005*
LAD^i^ (cm)	1.55 (1.21-1.99)	*<.001*	1.52 (1.16-1.98)	*.002*	1.45 (1.13-1.88)	*.003*
LVEF (%)	0.97 (0.96-0.98)	*<.001*	N/A^j^	N/A	N/A	N/A
LVEDD (mm)	N/A	N/A	1.20 (0.97-1.49)	.09	N/A	N/A
LVESD (mm)	N/A	N/A	N/A	N/A	1.39 (1.19-1.62)	*<.001*

^a^LVEF: left ventricular ejection fraction.

^b^LVEDD: left ventricular end-diastolic dimension.

^c^LVESD: left ventricular end-systolic dimension.

^d^HR: hazard ratio.

^e^Italic formatting represents *P* value<.05.

^f^SBP: systolic blood pressure.

^g^CCI: Charlson Comorbidity Index.

^h^AF: atrial fibrillation.

^i^LAD: left atrium dimension.

^j^N/A: not available.

### Determinants of TR Progression to 2 Grades or More

At a median follow-up of 4.6 (IQR 2.0-7.0) years, 282 out of 1081 patients had TR progression to greater than or equal to 2 grades or more. Univariate determinants of TR progression by 2 grades or more included age, female sex, CCI, the presence of AF, LVEF, statin use, and diuretics use (all *P*≤.02). Again, the telehealth group was not associated with TR progression to greater than or equal to 2 grades (HR 0.83, 95% CI 0.61-1.14; *P*=.25). Table 5 shows the final multivariate models for determinants of TR progression to 2 grades or more, which included older age, female sex, presence of AF, lower LVEF, and use of diuretics; statin use appeared to be protective.

**Table 5:** Multivariate determinants of tricuspid regurgitation progression in patients with less than or equal to mild-moderate tricuspid regurgitation at baseline: end point—increase of tricuspid regurgitation more than 2 grades (n=282).

	LVEF^a^ model			LVEDD^b^ model			LVESD^c^ model	
	HR^d^ (95% CI)	*P* value	HR (95% CI)		*P* value	HR (95% CI)		*P* value
Age	1.01 (1.00-1.02)	*.01* ^e^	1.00 (0.99-1.01)		.06	1.01 (1.00-1.02)		*.02*
Female	1.56 (1.19-2.05)	*.001*	1.46 (1.11-1.93)		*.007*	1.53 (1.16-2.02)		*.002*
SBP^f^	1.00 (0.99-1.00)	.35	1.00 (0.99-1.00)		.40	1.00 (0.99-1.00)		.37
CCI^g^	1.03 (0.94-1.12)	.43	1.03 (0.94-1.12)		.49	1.03 (0.94-1.12)		.46
AF^h^	2.18 (1.27-3.75)	*.009*	2.33 (1.36-3.99)		*.005*	2.31 (1.35-3.96)		*.005*
Diuretics	1.24 (0.95-1.63)	.10	1.39 (1.07-1.81)		*.01*	1.30 (0.99-1.71)		.05
Statin	0.76 (0.59-0.97)	*.03*	0.76 (0.59-0.98)		*.03*	0.77 (0.59-0.99)		*.04*
LAD^i^ (cm)	0.97 (0.78-1.21)	.80	1.01 (0.79-1.27)		.92	0.95 (0.76-1.19)		.69
LVEF (%)	0.98 (0.97-0.99)	*.006*	N/A^j^		N/A	N/A		N/A
LVEDD (mm)	N/A	N/A	0.94 (0.76-1.15)		.55	N/A		N/A
LVESD (mm)	N/A	N/A	N/A		N/A	1.12 (0.95-1.30)		.16

^a^LVEF: left ventricular ejection fraction.

^b^LVEDD: left ventricular end-diastolic dimension.

^c^LVESD: left ventricular end-systolic dimension.

^d^HR: hazard ratio.

^e^Italic formatting represents *P* value <.05.

^f^SBP: systolic blood pressure.

^g^CCI: Charlson Comorbidity Index.

^h^AF: atrial fibrillation.

^i^LAD: left atrium dimension.

^j^N/A: not available.

### Comparison Between Patients With and Without MR or TR Progression to Greater Than or Equal to Moderate

Patients with MR or TR progression from less than moderate to greater than or equal to moderate were older, more likely to be female, and had a higher prevalence of AF, larger baseline LA, LVEDD, LVSED, and lower LVEF as compared with those who did not progress to greater than or equal to moderate degree (Table 6).

**Table 6:** Baseline characters in those with and without progression of MR and TR to moderate or more.

	MR^a^ progression to greater than or equal to moderate				TR^b^ progression to greater than or equal to moderate		
	Yes	No	*P* value	Yes		No	*P* value
Age (years), mean (SD)	68 (14)	63 (14)	<.001	68 (14)		63 (14)	<.001
Female, n (%)	93 (39)	206 (24)	<.001	93 (39)		206 (24)	<.001
AF^c^ n (%)	14 (6)	19 (2)	.007	14 (6)		19 (2)	.007
LAD^d^ (cm), mean (SD)	3.9 (0.7)	3.7 (0.6)	<.001	3.9 (0.7)		3.7 (0.6)	<.001
LVEF^e^ (%), mean (SD)	58.3 (14.5)	64.1 (12.1)	<.001	58.3 (14.5)		64.1 (12.1)	<.001
LVEDD^f^ (mm)	4.9 (0.8)	4.7 (0.6)	.002	4.9 (0.8)		4.7 (0.6)	.002
LVESD^g^ (mm), mean (SD)	3.4 (0.9)	3.1 (0.7)	<.001	3.4 (0.9)		3.1 (0.7)	<.001

^a^MR: mitral regurgitation.

^b^TR: tricuspid regurgitation.

^c^AF: atrial fibrillation.

^d^LAD: left atrium dimension.

^f^LVEDD: left ventricular end-diastolic dimension.

^e^LVEF: left ventricular ejection fraction.

^g^LVESD: left ventricular end-systolic dimension.

### Comparison Between Patients With and Without Statin Use at Baseline

Table S1 in Multimedia Appendix 1 shows patients with and without baseline statin use. Those who used statins at baseline were more likely to be male, had less AF, more MI, used more antiplatelet, ACEI and ARB, nitrates, and beta-blockers (all *P*≤.02). Interestingly, despite more baseline history of MI, those who used statins at baseline did not suffer from more cardiac remodeling at follow-up.

## Discussion

### Overview

To our knowledge, this retrospective study, which included 1081 patients, is the first to explore the association between MR and TR progression and telehealth services. Our principal findings were as follows: (1) the telehealth group had higher burdens of comorbid conditions at baseline, including more heart failure and MI but similar cardiac remodeling indexes as compared with the control group; yet, the telehealth group did not exhibit higher prevalence of MR or TR progression; (2) determinants of MR progression to greater than or equal to moderate included age, female sex, dimensions of LA, LVEDD, LVESD, and LVEF; (3) determinants of TR progression greater than or equal to moderate included age, female sex, presence of AF, dimensions of LA, LVESD, and LVEF; and (4) statin use seemed to be protective for MR and TR progression.

### Effect of Telehealth Was Neutral for MR and TR Progression

Patients in our telehealth group had more comorbidities and were sicker at baseline but did not exhibit a higher prevalence of MR or TR progression as compared to the control group who were healthier individuals. This suggests that our telehealth services, which included remote monitoring of physiological parameters, timely clinical feedback, and more frequent care, may provide some kind of protection against MR and TR progression. Indeed, the lower baseline systolic BP of the telehealth group seems to indicate that better BP control was achieved through close monitoring of these patients. Further study is required to support this hypothesis.

The observation that our telehealth group was sicker was also seen in prior studies conducted by our telehealth center [15,16,19]; we believe that this could be due to the fact that patients with more comorbid conditions were more likely to pay for telehealth services.

### Factors Associated With MR Progression

We found that determinants of MR progression to greater than or equal to moderate degree included age, female sex, and dimensions of LA, LVEDD, LVESD, and LVEF. Avierinos et al [20], who defined MR progression as advancement by 1 grade or more, found that older age was independently associated with progression. Gomes et al [21] found that older age and larger LA volume were linked to MR progression. Additionally, patients with MR progression tended to have larger LV dimensions, LA volume, and lower LVEF at baseline, which aligns with the findings in this study (Table 6). This could be because cardiac chamber remodeling can lead to mitral valve tethering and enlarged mitral annulus [22], which in turn decreases mitral valve coaptation, resulting in more MR progression.

### Factors Associated With TR Progression

We found that determinants of TR progression were age, female sex, presence of AF, and dimensions of LA, LVESD, and LVEF. Enlarged LVESD indicates decreased intrinsic LV systolic function and is associated with reduced LVEF, while the presence of AF usually results in enlarged biatria. Our identified determinants for TR progression are largely consistent with previous studies. Song et al [23] investigated patients with persistent AF and found that patients with TR progression tended to be older and female; greater LA diameter was found to be an independent determinant of TR progression. In terms of gender differences, Gual-Capllonch et al [24] found that the prevalence of TR in females was higher and that AF was a specific independent determinant of TR progression in women. In a study somewhat similar to ours, Mutlak et al [25] found independent determinants of TR progression from trivial or mild to moderate or severe included age, female sex, AF, and LA enlargement. In particular, age and AF were strong determinants of TR progression. These findings are not surprising because up to 90% of TR is functional and caused by enlargement of the right atrium and right ventricle, as well as dilation of the tricuspid annulus [26].

### Medications and Progression of MR or TR

An interesting finding of our study was that statin use appeared to be protective of MR and TR progression, whereas diuretics had the opposite effect. In addition, those who used statins at baseline suffered from more MI and had higher ACEI and ARB use and beta-blocker use, and, interestingly, had similar cardiac chamber size (a marker of no progression of valvular heart disease) at follow-up as compared to those without baseline statin use. We suspect that the associations between statin use and less MR and TR progression could be the effects of more compliant guideline-directed medical treatment in the statin group. A previous study found that statin use was associated with slower progression of rheumatic mitral stenosis [27]. Interestingly, Varadarajan and Pai [28] found that in patients with severe AR, statin use was associated with a lower prevalence of TR at baseline. Whether statin use affects MR or TR progression warrants further investigations. We found it surprising that diuretics, which are commonly used for hypertension control or decongestion, were associated with a higher prevalence of MR or TR progression. As this was a retrospective study, there may be some confounders and uncollected factors, and we were also unable to verify drug compliance and adherence.

### Clinical Implications: How to Improve Telehealth Service and Move the Needle Toward Better Outcomes

Progression to significant MR or TR is associated with poor outcomes. Thus, periodic TTE surveillance, early detection, and timely intervention are mandatory for outcome improvement [29]. In a study conducted by Mutlak et al [25], progression from trivial or mild to moderate or severe TR was found to occur in 18.8% of patients over only 38 months of median follow-up, indicating that TR may progress rapidly. In real-world scenarios and busy clinical practices, timely monitoring of TTEs may be a barrier, as patients need to be evaluated by clinicians before they can be scheduled for a TTE exam, which often takes place several weeks later.

The importance of imaging as a diagnostic tool for MR and TR progression should not be ignored. In their commentary, Narula et al [30] highlighted the key role of bedside ultrasounds as the “fifth pillar” in physical examinations. However, advances in diagnostic technology mean that handheld cardiac ultrasound probes such as point-of-care ultrasound (POCUS) [31] are now available for at-home use and can be paired with emerging technologies and preliminary training to enhance novice home-based usage [32,33], meaning that even patients who have not undergone professional medical training can easily obtain echocardiograms to assess LA and LV size and LVEF in the comfort of their own homes. Promotion of this and similar devices can facilitate frequent monitoring of symptoms and parameters associated with MR or TR progression as found in this study.

Currently, the telehealth services offered by NTUH only include monitoring of BP, blood sugar, oxygen saturation, and 1-lead electrocardiogram and do not provide parameters associated with MR and TR progression. TTEs are not routinely ordered as they are inconvenient and time-consuming; yet, cardiac size and function are paramount in determining the future progression of MR and TR. Therefore, to improve the outcomes of telehealth patients, who tend to be sicker as we have shown herein, we recommend the following: (1) POCUS in tandem with cutting-edge artificial intelligence technology should be promoted for at-home use by patients or their caregivers. Relevant parameters such as LV/LA size and LVEF can be uploaded to telehealth platforms and alert notifications be sent to the case managers or physicians. Measurement of inferior vena cava to assess degrees of fluid overload for timely diuretic dosage adjustment may also improve outcomes; (2) the detection of AF during telehealth monitoring should be regarded as a red flag by case managers as it is linked to TR progression; (3) special care and additional monitoring should be provided for female patients. The abovementioned indexes can be highlighted in telehealth platforms in order to provide customized care for our patients.

### Limitations

This was a retrospective observational study, which used data taken from a tertiary-referral center, and we were, therefore, unable to eliminate the potential for selection bias. No information was included on MR or TR mechanisms. TR and MR grades were assessed by eyeball as quantification was not routinely implemented in NTUH prior to 2021. We were unable to obtain detailed information regarding the medication compliance of patients.

### Conclusions

Patients undergoing telehealth interventions who were sicker at baseline were found to exhibit a similar prevalence of MR or TR progression compared to a control group. Determinants of MR and TR progression included easy-to-measure traditional echo parameters of cardiac function, older age, female sex, and presence of AF, which could be incorporated into a telehealth platform and alerting system to improve patient outcomes through personalized care. In this current era of precision medicine, the promotion of POCUS use in telehealth care seems reasonable and feasible, especially considering the technological advances that can be used to aid novice users.

## Appendix

Multimedia Appendix 1 Data transmission process and characteristics of patients stratified by the use of statin.

## Abbreviations

ACEI: angiotensin-converting enzyme inhibitor
AF: atrial fibrillation
ARB: angiotensin receptor blocker
BP: blood pressure
CCI: Charlson Comorbidity Index
HR: hazard ratio
LA: left atrial
LVEDD: left ventricular end-diastolic dimension
LVEF: left ventricular ejection fraction
LVESD: left ventricular end-systolic dimension
MI: myocardial infarction
MR: mitral regurgitation
NTUH: National Taiwan University Hospital
POCUS: point-of-care ultrasound
TR: tricuspid regurgitation
TTE: transthoracic echocardiogram
