# Correction: Effect of Telehealth Services on Mitral and Tricuspid Regurgitation Progression: Retrospective Study

**DOI:** 10.2196/53232

**Published:** 2023-10-04

**Authors:** Li-Tan Yang, Jen-Kuang Lee, Chieh-Mei Tsai, Ying-Hsien Chen, Ching-Chang Huang, Hui-Wen Wu, Chin-Hua Su, Chien-Chang Lee, Chi-Sheng Hung, Yi-Lwun Ho

**Affiliations:** 1 Division of Cardiology, Department of Internal Medicine National Taiwan University Hospital Taipei Taiwan; 2 Department of Internal Medicine College of Medicine National Taiwan University Taipei Taiwan; 3 Telehealth Center National Taiwan University Hospital Taipei Taiwan; 4 Department of Emergency Medicine National Taiwan University Hospital Taipei Taiwan; 5 Division of Cardiology Department of Internal Medicine National Taiwan University Hospital Taipei Taiwan

In “Effect of Telehealth Services on Mitral and Tricuspid Regurgitation Progression: Retrospective Study” (J Med Internet Res 2023;25:e47947) the authors noted one error.

The third author’s name was listed as:

Chie-Mei Tsai

This has been changed to the following:

Chieh-Mei Tsai

The correction will appear in the online version of the paper on the JMIR Publications website on October 4, 2023 together with the publication of this correction notice. Because this was made after submission to PubMed, PubMed Central, and other full-text repositories, the corrected article has also been resubmitted to those repositories.

