# Supplementary material for: Effect of Telehealth Services on Mitral and Tricuspid Regurgitation Progression: Retrospective Study
Source: J Med Internet Res. 2023 Sep 26;25:e47947. doi: 10.2196/47947 (PMC10565617; doi:10.2196/47947)
Supplement: Multimedia Appendix 1 [file jmir_v25i1e47947_app1.docx]

**Figure S1.** The data transmission process and subsequent handling by our Telehealth Center.

**
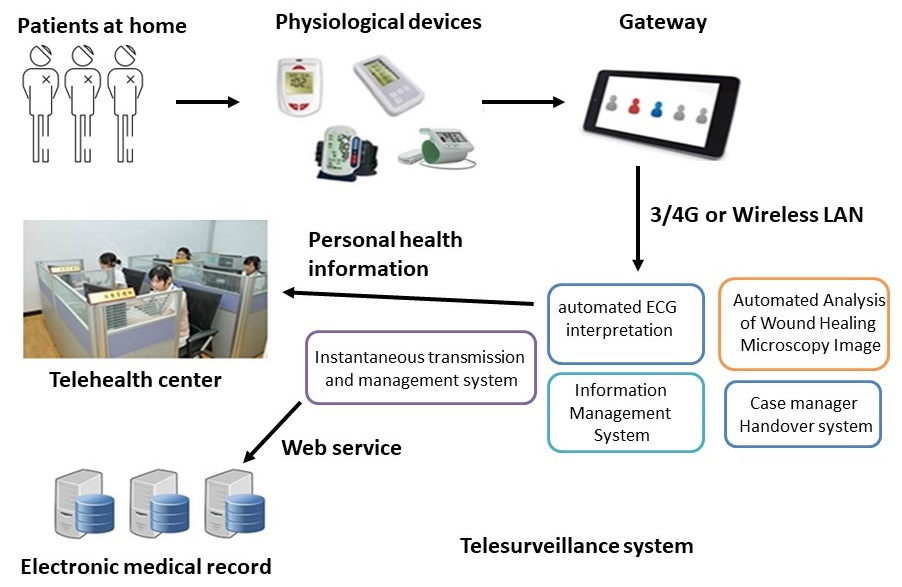
**

**Table S1.** Characteristics between patients with and without baseline statin use.

|  | **Statin**  **(n=542)** | **Non-statin**  **(n=539)** | P value |
| --- | --- | --- | --- |
| Age | 63±12 | 64±16 | .33 |
| Male | 417(77) | 365(68) | **<.001** |
| SBP, mmHg | 131±20 | 131±21 | .90 |
| DBP, mmHg | 75±13 | 74±14 | .29 |
| Atrial fibrillation | 10(2) | 23(4) | **.02** |
| Charlson comorbidity index | 1.19±1.47 | 1.14±1.51 | .63 |
| Hypertension | 282(52) | 263(49) | .28 |
| Diabetes mellitus | 167(31) | 141(26) | .09 |
| Myocardial infarction | 80(15) | 43(8) | **<.001** |
| Heart failure history | 66(12) | 85(16) | .08 |
| Malignancy | 37(7) | 41(8) | .62 |
| Telehealth group | 113(21) | 113(21) | .96 |
| **Medications** |  |  |  |
| Antiplatelet | 488(90) | 307(57) | **<.001** |
| Alpha blocker | 69(13) | 73(14) | .69 |
| ACEi/ARB | 352(65) | 263(49) | **<.001** |
| Beta blocker | 416(77) | 308(57) | **<.001** |
| Calcium channel blocker | 216(40) | 214(40) | .96 |
| Diuretics | 216(40) | 194(36) | .19 |
| Nitrate | 289(53) | 210(39) | **<.001** |
| **Baseline cardiac chamber size** |  |  |  |
| LVEF, % | 62±12 | 63±13 | .15 |
| LA dimension, cm | 3.8±0.6 | 3.8±0.6 | .78 |
| LVEDD, mm | 48±6 | 48±7 | .68 |
| LVESD, mm | 32±8 | 32±8 | .56 |
| **Baseline MR** |  |  | **.04** |
| None | 41(8) | 54(10) |  |
| Trivial | 44(8) | 39(7) |  |
| Mild | 382(70) | 344(64) |  |
| Mild-moderate | 75(14) | 102(19) |  |
| **Baseline TR** |  |  | .23 |
| None | 54(10) | 47(9) |  |
| Trivial | 64(12) | 55(10) |  |
| Mild | 361(67) | 353(65) |  |
| Mild-moderate | 63(12) | 84(16) |  |
| **Maximal MR during follow-up** |  |  | .40 |
| None | 7(1) | 7(1) |  |
| Trivial | 12(2) | 12(2) |  |
| Mild | 305(56) | 280(52) |  |
| Mild-moderate | 113(21) | 108(20) |  |
| Moderate | 92(17) | 121(22) |  |
| Moderate-severe | 10(2) | 10(2) |  |
| Severe | 3(<1) | 1(<1) |  |
| **Maximal MR ≥moderate** | 105(19) | 132(24) | **.04** |
| **Maximal MR ≥2 grades compared to baseline** | 114(21) | 139(26) | .06 |
| LA dimension at maximal MR, cm | 4.0±0.6 | 4.0±0.7 | .96 |
| LVEF at maximal MR, % | 63±13 | 63±13 | .51 |
| LVEDD at maximal MR, mm | 49±6 | 49±7 | .62 |
| LVESD at maximal MR, mm | 33±8 | 32±9 | .57 |
| **Maximal TR during follow-up** |  |  | **.001** |
| None | 3(<1) | 2(<1) |  |
| Trivial | 10(2) | 5(<1) |  |
| Mild | 316(58) | 263(49) |  |
| Mild-moderate | 108(20) | 115(21) |  |
| Moderate | 95(18) | 131(24) |  |
| Moderate-severe | 7(1) | 22(4) |  |
| Severe | 3(<1) | 1(<1) |  |
| **Maximal TR ≥moderate** | 105(19) | 132(24) | **.04** |
| **Maximal TR ≥2 grades compared to baseline** | 126(23) | 156(29) | **.03** |
| LA dimension at maximal TR, cm | 4.0±6 | 4.0±7 | .66 |
| LVEF at maximal TR, % | 63±13 | 63±13 | .69 |
| LVEDD at maximal TR, mm | 49±6 | 49±7 | .48 |
| LVESD at maximal TR, mm | 32±8 | 32±9 | .58 |

LVEF, Left ventricular ejection fraction. See other abbreviations from Table 1.
